# Neuroprotective effect of tangeretin against chromium-induced acute brain injury in rats: targeting Nrf2 signaling pathway, inflammatory mediators, and apoptosis

**DOI:** 10.1007/s10787-023-01167-3

**Published:** 2023-03-08

**Authors:** Ahmed A. Sedik, Rania Elgohary

**Affiliations:** 1 Pharmacology Department, Medical Research and Clinical Studies Institute, National Research Center, El-Buhouth St., Dokki, Cairo, 12622 Egypt; 2 Narcotics, Ergogenics and Poisons Department, Medical Research and Clinical Studies Institute, National Research Center, Cairo, 12622 Egypt

**Keywords:** Tangeretin, Inflammation, Nrf2, Caspase-3, Chromium, Brain Injury

## Abstract

Potassium dichromate (PD) is an environmental xenobiotic commonly recognized as teratogenic, carcinogenic, and mutagenic in animals and humans. The present study was conducted to investigate the role of tangeretin (TNG) as a neuro-protective drug against PD-induced brain injury in rats. Thirty-two male adult Wistar rats were blindly divided into four groups (8 rats/group). The first group received saline intranasally (i.n.). The second group received a single dose of PD (2 mg/kg, i.n.). The third group received TNG (50 mg/kg; orally), for 14 days followed by i.n. of PD on the last day of the experiment. The fourth group received TNG (100 mg/kg; orally) for 14 days followed by i.n. of PD on the last day of the experiment. Behavioral indices were evaluated 18 h after PD administration. Neuro-biochemical indices and histopathological studies were evaluated 24 h after PD administration. Results of the present study revealed that rats intoxicated with PD induced- oxidative stress and inflammation via an increase in malondialdehyde (MDA) and a decrease in nuclear factor erythroid 2-related factor 2 (Nrf2) signaling pathway and glutathione(GSH) levels with an increase in brain contents of tumor necrosis factor-alpha (TNF-α) and interleukin (IL-6). Pre-treatment with TNG (100 mg/kg; orally) ameliorated behavior, cholinergic activities, and oxidative stress and decreased the elevated levels of pro-inflammatory mediators; TNF-α and IL-6 with a decrease in brain content of chromium residues detected by Plasma–Optical Emission Spectrometer. Also, the histopathological picture of the brain was improved significantly in rats that received TNG (100 mg/kg). Additionally, TNG decreased caspase-3 expression in the brain of PD rats. In conclusion, TNG possesses a significant neuroprotective role against PD-induced acute brain injury via modulating the Nrf2 signaling pathway and quenching the release of inflammatory mediators and apoptosis in rats.

**Graphical abstract:**

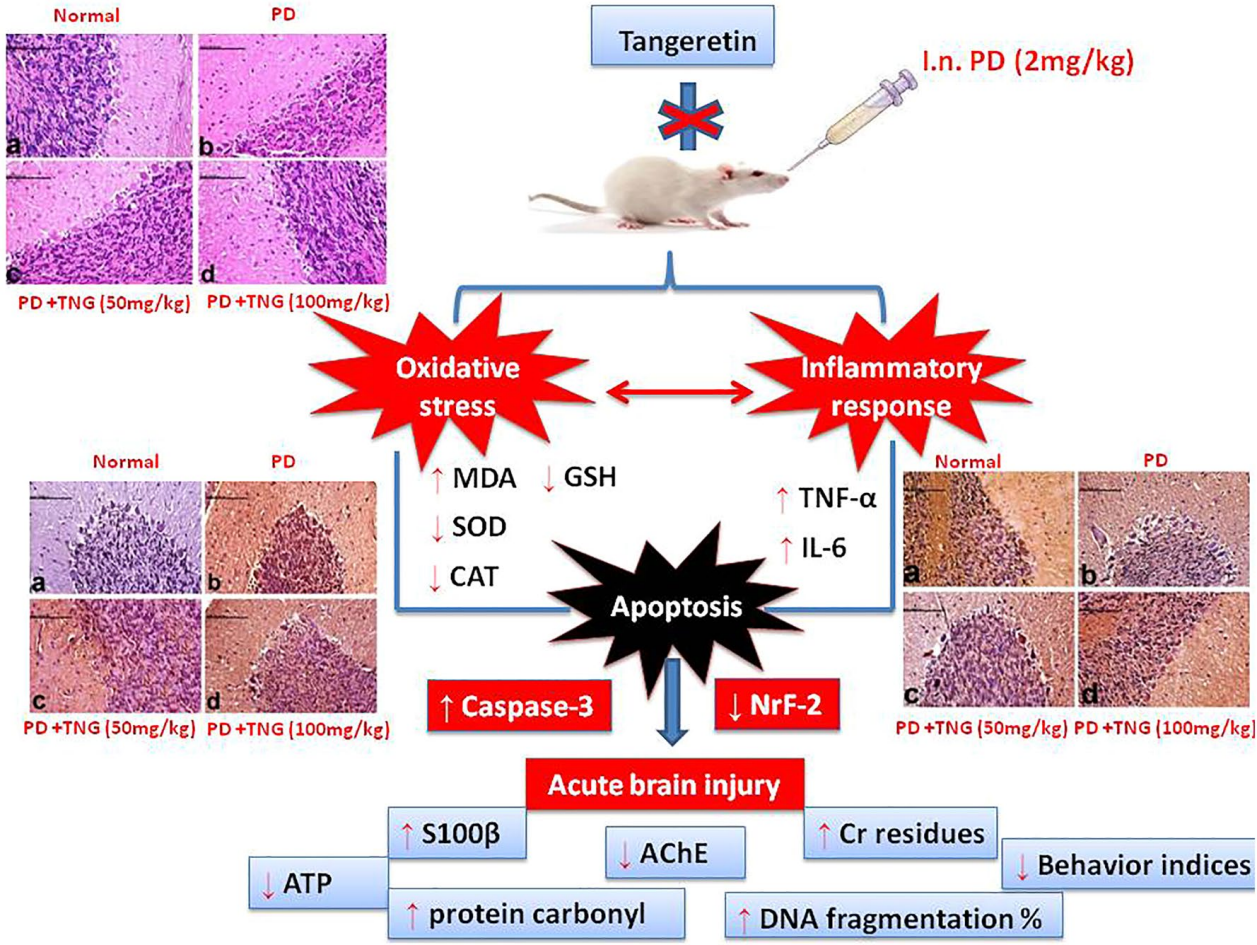

## Introduction

Heavy metal exposure is one of the most important environmental problems and has terrible consequences for human health (Luo et al. 2021). The biological effects of heavy metal toxicity are correlated with their chemical characteristics. The serious complications associated with such heavy metals, especially neurotoxicity, have recently attracted the attention of researchers (Khan and Parvez 2015).

Chromium (Cr) is an environmental heavy metal that accidently presents in different industrial processes (Tchounwou et al. 2012). According to a report published by the International Agency for Research on Cancer (IARC) in 2018, Cr (VI) has been categorized as a group I occupational carcinogen (Loomis et al. 2018). Potassium dichromate (K2Cr2O7) is considered the highly hazardous among Cr (VI) salts. K2Cr2O7 has been extensively employed in a range of industrial applications such as construction and leather manufacturing (Bregnbak et al. 2015).

Brain injury is a concern in global public-health problem, and a death-leading cause worldwide. Brain injury raises the risk of neurodegenerative diseases and is a major cause of life-long disability (Shaver et al. 2019). Long-term problems in the cognitive, physical and emotional domains are a result of secondary impairments from brain injury (Smith et al. 2022). Such impairments are often discribed as the “silent epidemic”. People under 40 years, it culminated in 150–200 people per million being disabled annually. While millions of people with brain injuries receive emergency medical attention each year, 1.5 million people each year pass away (De Silva et al. 2008). The brain is usually susceptible to intoxication with Cr, he unsaturated fat cells in the brain, subjected it  to have a weak antioxidant defense system, making it more susceptible to reactive oxygen species (ROS) and reactive nitrogen species (RNS), leading to oxidative damage in cellular DNA, lipids and proteins, which end with inflammatory processes, cell death or apoptosis (Casalegno et al. 2015; Hao et al. 2017).

Nuclear factor erythroid 2-related factor 2 (Nrf2), has a cytoprotective and neuroprotective response by regulating different antioxidant genes. Recent studies have demonstrated the upregulation of Nrf2 as an endogenous defense response in brain injury (Sharma et al. 2020). Apoptosis is a genetically controlled cell suicide program that has a significant impact on the pathophysiology of brain damage and results in internucleosomal DNA fragmentation. Activation of caspase-3 is a well-known characteristic of neuronal apoptosis in numerous central nervous system (CNS) disorders and this pathway is considered a promising therapeutic target (Macks et al. 2022). Furthermore, one of the key factors leading to caspase-3-mediated apoptosis pathways is oxidative stress (Xu et al. 2022).

Cytokines are released after brain injury and become entangled in behavioral reactions (cognitive and motor reactions) (Bailey et al. 2006). After a brain injury, pro-inflammatory and cytotoxic factors are released that damage neurons and further activate microglia, causing gradual degeneration in dopaminergic neurons that control motor and cognitive functions (Salem et al. 2022). The neuroprotective effects of natural substances in brain injury have recently attracted a lot of attention.

Tangeretin (TNG), a polymethoxylated flavone, is widely distributed in citrus species' peel tissues, including *citrus tangerine* and *citrus depressa*. Numerous pharmacological benefits of TNG, including neuroprotective, anti-cancer, anti-inflammatory, anti-oxidative, and anti-diabetic actions, have been shown. According to several studies, TNG lessens cognition and memory deficits by reducing neuroinflammation in experimental animals (Chen et al. 2022). We postulated that activation of the anti-oxidant pathways and inhibition of inflammatory and pro-apoptotic mediators could protect the brain against chromium-induced brain injury. Given this background, the current study was conducted to study the potential protective effect of TNG and its possible mechanisms through stimulation of the Nrf2 signaling pathway against chromium-induced brain injury.

## Materials and methods

### Animals

Adult male Wistar albino rats weighing 150–170 g were provided from the colony of the National Research Centre (NRC), Giza, Egypt. Rats were kept under temperature (21 ± 1 °C), humidity (60%), and 12 h light/12 h dark photoperiod, with free access to feed and water. The study was approved by the National Research Centre’s Medical Research Ethics Committee (MREC) (42,311,122,022). Furthermore, the research protocol adhered to the National Institutes of Health's guidelines for the care and use of laboratory animals. (Garber et al. 2011). The present animal experiment adhered to the ARRIVE guidelines (Du Sert et al. 2020).

### Chemicals

Potassium dichromate (PD, CAS No: 7778–50-9/purity, 99%), and tangeretin (TGN, CAS No: 481–53-8/purity, 99%) were obtained from Sigma–Aldrich, USA, whereas other reagents and chemicals were of the highest grade available.

#### Induction of acute brain injury

Acute brain injury was induced via, single intranasal (i.n.) administration of (PD; 2 mg/kg) in rats as it mimics the pathophysiological features of brain injury in humans (Salama and Elgohary 2021).

### Study design (Fig. 1)

**Fig. 1:**
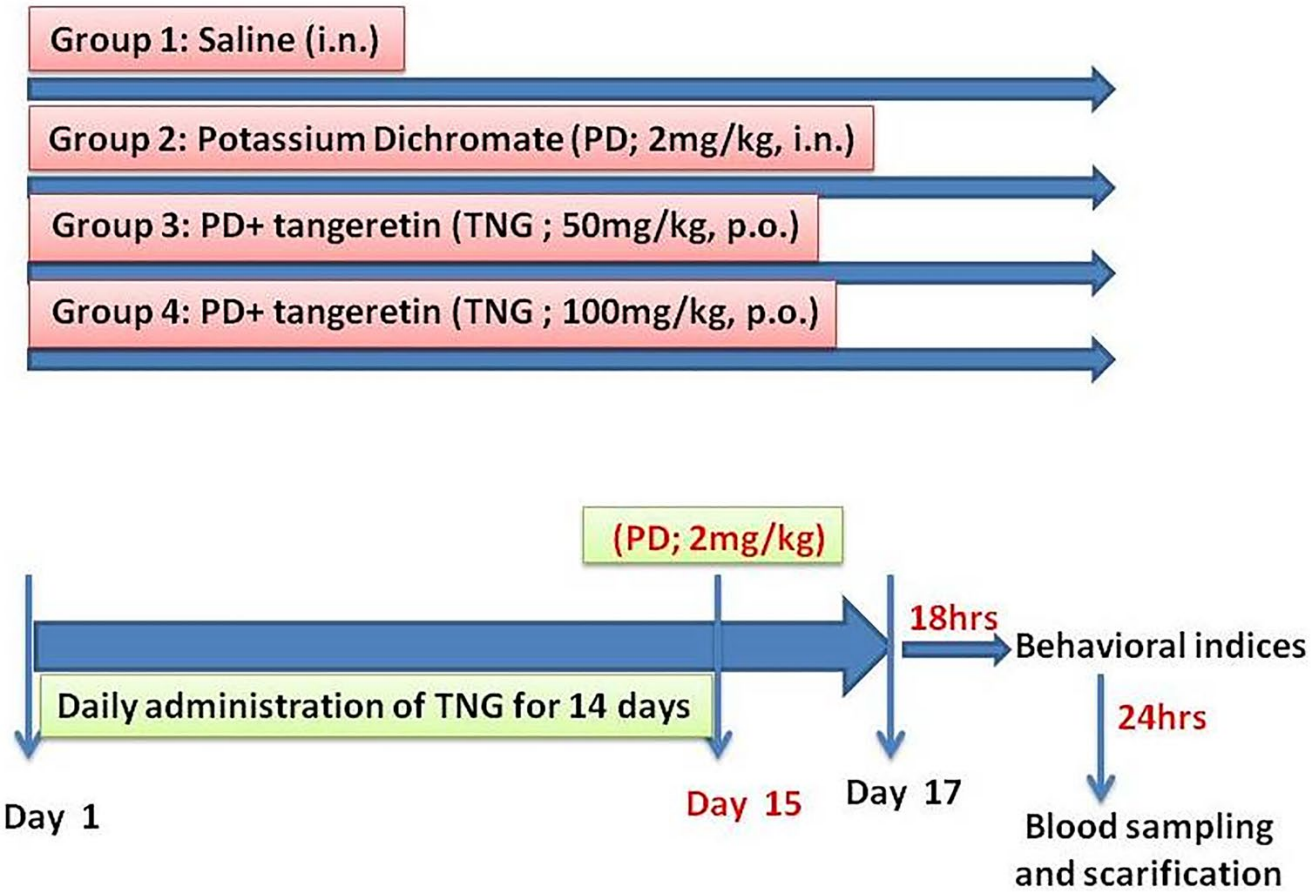
SchemaLtic representation of the experimental design

Thirty-two male Wistar albino rats were blindly divided into four groups (8 rats/group). The first group received saline (i.n.) thought the study. The second group received PD (2 mg/kg, i.n.)**.**The third group received TNG (50 mg/kg; orally), for 14 days followed by i.n. administration of PD. The fourth group received TNG (100 mg/kg; orally) for 14 days followed by i.n. injection of PD on the last day of the experiment (Omar et al. 2016). 18 h after administration of PD, behavioral indices were evaluated as follows:

### Behavioral indices

Motor activity and motor coordination were measured 18 h after the last dose of PD administration.

#### Evaluation of motor activity

Motor activity was evaluated with the aid of an activity cage depending on the infrared photocell principle (Ugo-Basile, Model 7430, Italy). Before, placing the rats in the cage, they were adapted to the test room for 1 h daily for 3 training sessions (5 min/session). At the end of the experiment (18 h after the last dose of PD), motor activity for each rat was scored over 5 min **(**Afifi et al. 2021**).**

#### Evaluation of motor coordination

Motor coordination was conducted according to the method previously described by Vijitruth et al. (2006). Before starting the training session, rats were habituated freely on the accelerating rotarod (Ugo Basile, Model 7750, Italy). 18 h after the last dose of PD, rats were re-placed again to count the motor performance over 5 min (Salama and Ibrahim 2015).

#### Open field test

Ambulatory and locomotor activities of rats were evaluated using an open field test by utilizing a wooden box (90 × 90 × 50 cm high), divided into nine squares and covered with a layer of white impermeable formica for the ease of cleaning and durability (Yu et al. 2017). Rats were given 15 min to roam the arena. On the testing day; each rat was positioned centrally and the count number of squares crossed with at least three paws (crossing) and the standings on the hind legs (rearing) were evaluated (Lanznaster et al. 2017).

### Neuro-biochemical markers

#### Blood sampling and preparation of brain homogenate

Blood was drawn from the eye’s retro-orbital plexus of the rats after being anesthetized with ketamine (100 mg/kg) and xylazine (10 mg/kg). Using a cooling centrifuge, Sera were separated by centrifugation at 3000 rpm for 15 min at 4 °C (Laborezentrifugen, 2k15, Sigma, Germany**)**. Rats were decapitated under light anesthesia, and their brains were dissected, washed with saline, and divided into two parts. Part 1 was immersed in ice-cold phosphate buffer (pH 7.4) to prepare a 20% homogenate with a tissue homogenizer (MPW-120, BitLab Medical instruments, Poland). Using a cooling centrifuge, homogenized tissues were centrifuged at 4000 rpm/min for 10 min at 4 C. (Laboratory Centrifuge, 2 K15, Sigma Co., Germany) (Sedik and Hassan 2022). The supernatant was gathered, kept at -80 °C, and used to estimate additional biochemical indices. Regarding part 2, the cerebellum was prepared for histopathological and immunohistochemical analyses of the levels of caspase-3 and Nrf2.

#### Serum level of rat soluble protein-100β (S100β) in serum

Serum levels of S100β were measured with the aid of a quantitative sandwich enzyme immunoassay method using a commercial kit (CUSABIO, Cat. No: CSB- E08066r, China) (Leite et al. 2008).

#### Evaluation of acetylcholinesterase (AChE) and total adenosine triphosphate (ATP) levels

The activity of AChE in brain homogenate was measured, depending on a reaction between thiocholine and dithiobisnitrobenzoate (Ellman et al. 1961). The levels of ATP in brain samples were measured at wavelength 450 nm by using an ELISA kit (Cat. No: KT-59182, Kamiya Biomedical Co.) (Cailla et al. 1982).

#### Determination of malondialdehyde (MDA) and reduced glutathione (GSH)

The concentrations of MDA and GSH were detected in brain homogenates spectrophotometrically at wavelength 535 nm and 412 nm, respectively (Beutler 1969; Nair and Turner 1984).

#### Determination of catalase (CAT) and superoxide dismutase (SOD)

Evaluation of CAT and SOD in brain homogenate was performed by a spectrophotometric method developed by (Luck 1965) and (Sun et al. 1988), respectively.

#### Determination of protein carbonyl levels in brain tissue

The concentration of protein in brain homogenate was determined chemically by using bovine serum albumin as standard (Levine et al. 1990).

#### DNA fragmentation assay for brain tissue

The DNA fragmentation assay is a method for quantifying DNA damage (Perandones et al. 1993). After being lysed in 0.5 mL of hypotonic lysis buffer (10 mmol/L Tris–HCl pH 8), 1 mmol/L EDTA, and 0.2% triton X-100, brain samples were centrifuged at 14000×*g* for 20 min at 4 °C. The pellets were resuspended in a lysis buffer that is hypotonic. 0.5 mL of 10% trichloroacetic acid (TCA) was added to the supernatants and the pellets after they had been resuspended. The pellets were suspended in 5% TCA after the samples were centrifuged for 20 min at 10,000×*g* at 4 °C. Each sample was then given a double dose of diphenylamine and allowed to sit at 4 °C for 48 h. At 578 nm, the optical density (OD) was last measured. The following equation was used to determine the percentage of DNA fragmentation:

$${\text{DNA fragmentation}}\,\%\, =\, {\text{OD of supernatant}}/ \, ({\text{OD of supernatant}} + {\text{OD of pellet}}) \, \times { 1}00.$$

#### Determination of Cr residues in brain tissue

Lyophilized brain samples were digested and prepared according to a standard analytical method **(**Rice et al. 2012**)**. Analysis of Cr residues in brain tissues was performed by Agilent 5100 Inductively Coupled Plasma–Optical Emission Spectrometer (*ICP-OES*) with Synchronous Vertical Dual View (SVDV). For each series of measurement intensity, a calibration curve was constructed that was composed of a blank and three or more standards from Merck, Germany.

#### Determination of TNF-α and il-6 in brain tissue

TNF-α and il-6 levels were assessed in brain homogenate by using the rat TNF-α ELISA kit (Sunlong Biotech Co., Catalog no. SL0722Ra, CHINA) and il-6 ELISA Kit (Sunlong Biotech Co., Catalog no. SL0411Ra, CHINA), depending on the sandwich-ELISA method. The optical density (OD) for the concentration TNF-α and il-6 were measured spectrophotometrically at a wavelength of 450 nm (Grellner et al. 2000).

## Statistical analysis

One-way analysis of variance (ANOVA) was used for all quantifiable comparisons in our study, and Tukey's multiple comparison tests were performed using the GraphPad Prism program 8.0, USA). Results are presented as mean ± SEM of Eight rats and the difference was documented as significant when the *p* value is ≤ 0.05.

## Results

### Effect of tangeretin on the values of Cr residues in the brain of rats received PD induced—acute brain injury

PD induced—acute brain injury showed a significant elevation (*p* < 0.05) by 23-fold in the values of brain Cr residue in the PD-intoxicated group, after intranasal exposure. While, treatment with TNG 50 and 100 mg significantly (*p* < 0.05) decreased the Cr residue level by 57 and 99% respectively, as compared to the PD treated rats (Table 1).

**Table 1:** Effect of tangeretin on the values of chromium residues in the brain of rats received PD induced—acute brain injury

Groups	Normal	PD	PD + TNG (50 mg/kg)	PD + TNG (100 mg/kg)
Chromium residues	0.03 ± 0.01 mg/kg	7 ± 0.03 mg/kg^a^	3 ± 0.03 mg/kg^ab^	0.08 ± 0.01 mg/kg^bc^

Pre-treatment of acute brain injury induced by i.n dose of PD with TNG orally either 50 or 100 mg/kg for 14 days. Values of Cr residues in the brain were measured 24 h after the last dose of the drug. Results are expressed as mean ± SEM (*n* = 8)

^a^Significant difference from normal group *p* < 0.05

^b^Significant difference from PD group *p* < 0.05

^c^Significant difference from PD + TNG (50 mg/kg) group *p* < 0.05

### Effect of tangeretin on motor activity, motor coordination, and open field test (rearing & crossing) in rats received PD induced—acute brain injury

**Fig. 2:**
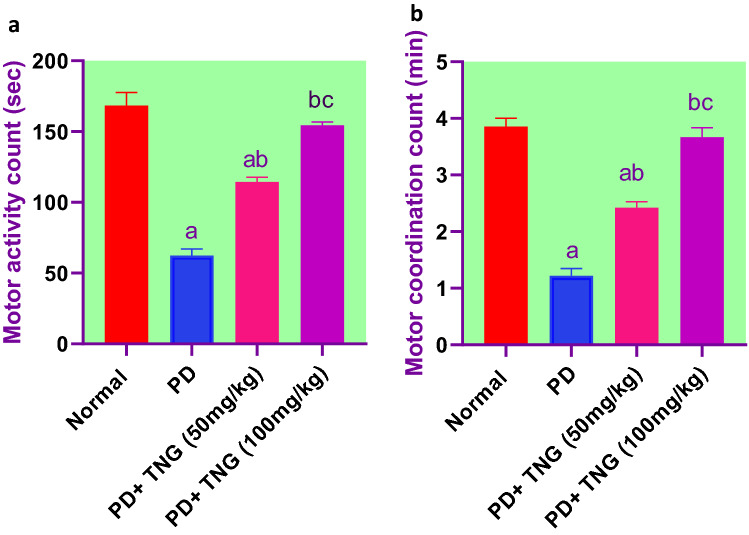
Effect of tangeretin on motor activity and motor coordination in rats received PD induced - acute brain injury. Pre-treatment of acute brain injury induced by i.n dose of PD with TNG orally either 50 or 100 mg/kg for 14 days. (**a**) Motor activity; (**b**) Motor coordination were measured 18h after the last dose of the drug. Results are expressed as mean ± SEM (*n* = 8). ^a^Significant difference from normal group *P* < *0.05*. ^b^Significant difference from PD group *P* < *0.05*. ^c^Significant difference from PD+TNG (50 mg/kg) group *P* < *0.05*

PD administration revealed a marked reduction in motor activity and coordination by 63 and 69%, respectively, as compared to normal control (*p* < 0.05). While, TNG treatment at 50 and 100 mg significantly (*p* < 0.05) improved the performance, by elevation of motor activity and coordination by 83.4%, 148%, and 100%, 202% respectively, as compared to the PD group. Moreover, TNG 100 mg/kg returned both to a normal value (Fig. 2).

**Fig. 3:**
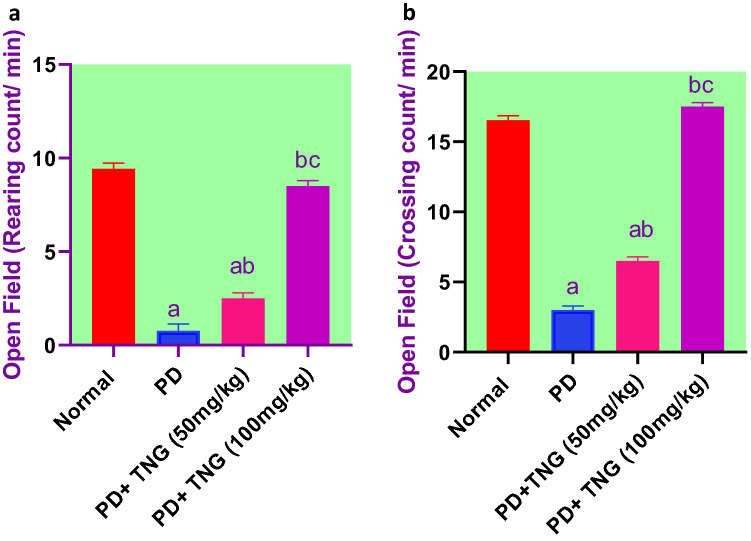
Effect of tangeretin on open field test (rearing & crossing) in rats received PD induced - acute brain injury. Pre-treatment of acute brain injury induced by i.n dose of PD with TNG orally either 50 or 100 mg/kg for 14 days. (**a**) Rearing; (**b**) Cossing counts were measured 18h after the last dose of the drug. Results are expressed as mean ± SEM (n = 8). ^a^Significant difference from normal group *P* < *0.05*. ^b^Significant difference from PD group *P* < *0.05*. ^c^Significant difference from PD+TNG (50 mg/kg) group *P* < *0.05*

Rats that received PD have shown a significant (*p* < 0.05) decrease in the rearing count by 92% and the number of squares crossed by 82%, respectively, as compared to the control group rats. On the other hand, TNG 50 and 100 mg treated rats showed a significant increase (*p* < 0.05) in the rearing count, and the number of squares crossed by 229%, one-fold, and 117%, 483%, respectively, as compared to the PD treated group. Furthermore, TNG (100 mg/kg) could restore it to normal values (Fig. 3).

### Effect of tangeretin on the serum levels of S100β in rats received PD induced—acute brain injury

**Fig. 4:**
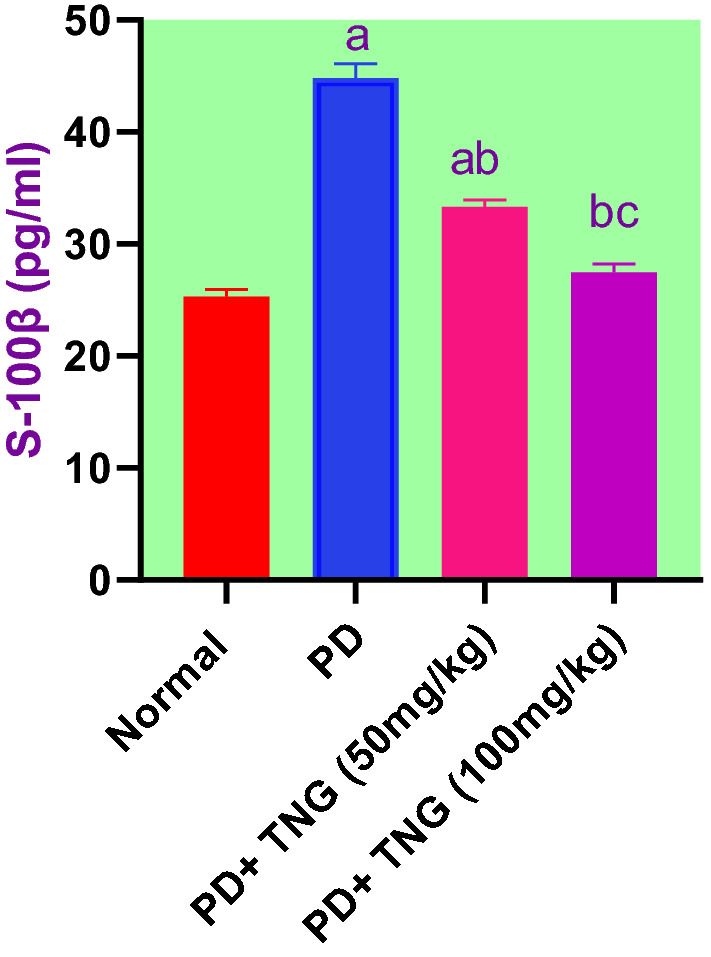
Effect of tangeretin on the serum levels of S100β in rats received PD induced - acute brain injury. Pre-treatment of acute brain injury induced by i.n dose of PD with TNG orally either 50 or 100 mg/kg for 14 days. Serum levels of S100β were evaluated 24h after the last dose of the drug. Results are expressed as mean ± SEM (n = 8). ^a^Significant difference from normal group *P* < *0.05*. ^b^Significant difference from PD group *P* < *0.05*. ^c^Significant difference from PD+TNG (50 mg/kg) group *P* < *0.05*

S100β was markedly increased by 77% after acute brain injury induced in the serum of PD rats when compared to the control group rats While S100β levels in the TNG 50 and 100 mg treatment groups were significantly (*p* < 0.05) lowered by 26 and 39% respectively, as compared to PD treated group. Moreover, TNG (100 mg/kg) could restore the normal values of S100β (Fig. 4).

### Effect of tangeretin on the levels of AChE and ATP in rats received PD induced—acute brain injury

**Fig. 5:**
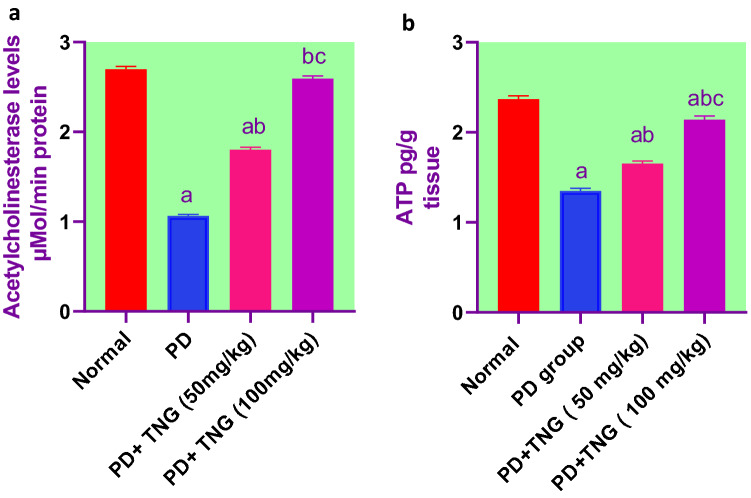
Effect of tangeretin on AChE and ATP levels in rats received PD induced - acute brain injury. Pre-treatment of acute brain injury induced by i.n dose of PD with TNG orally either 50 or 100 mg/kg for 14 days. (**a**) AChE; (**b**) ATP levels were measured 24h after the last dose of the drug. Results are expressed as mean ± SEM (n = 8). ^a^Significant difference from normal group *P* < *0.05*. ^b^Significant difference from PD group *P* < *0.05*. ^c^Significant difference from PD+TNG (50 mg/kg) group *P* < *0.05*

Brain AChE and ATP levels were significantly (*p* < 0.05) decreased in PD group by 61 and 43%, respectively, as compared to the control group. Meanwhile, TNG 50 and 100 mg administration significantly (p < 0.05) improved the decreased levels of the Brain AChE by 64%, 144%, and ATP levels by 22% and 59% respectively, as compared to PD group. Moreover, TNG 100 mg/kg returned them to a normal value (Fig. 5).

### Effect of tangeretin on oxidative stress indices in rats received PD induced—acute brain injury

**Fig. 6:**
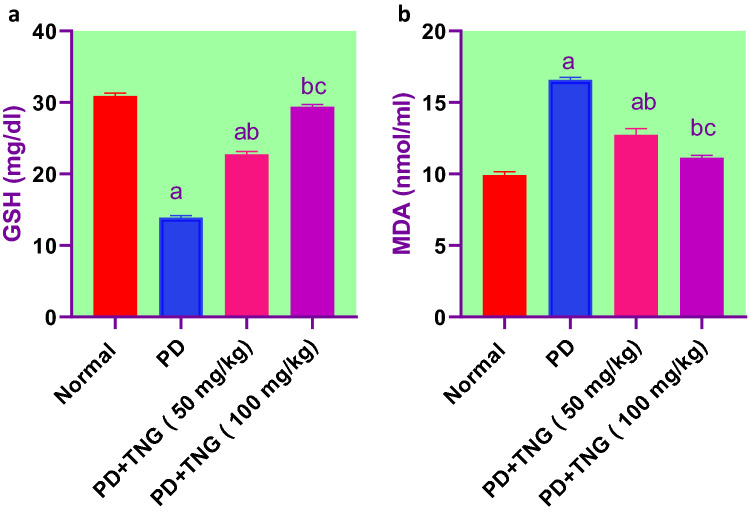
Effect of tangeretin on GSH and MDA levels in rats received PD induced - acute brain injury. Pre-treatment of acute brain injury induced by i.n dose of PD with TNG orally either 50 or 100 mg/kg for 14 days. (**a**) GSH; (**b**) MDA levels were measured 24h after the last dose of the drug in brain homogenate. Results are expressed as mean ± SEM (n = 8). ^a^Significant difference from normal group *P* < *0.05*. ^b^Significant difference from PD group *P* < *0.05*. ^c^Significant difference from PD + TNG (50 mg/kg) group *P* < *0.05*

**Fig. 7:**
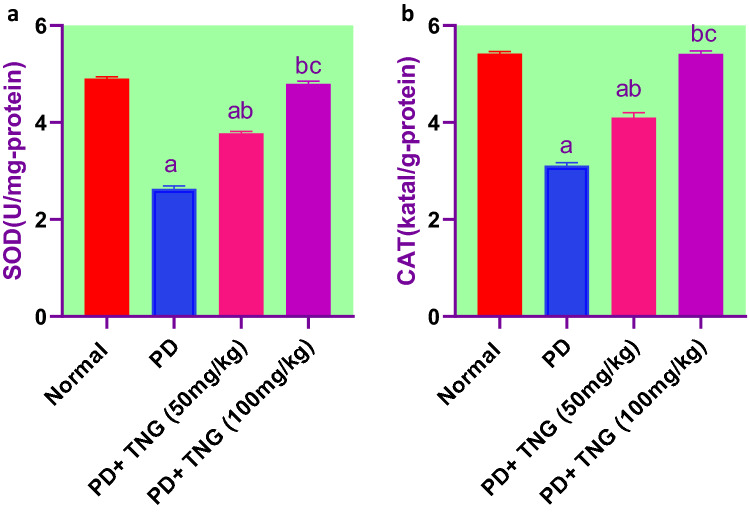
Effect of tangeretin on SOD and CAT levels in rats received PD induced - acute brain injury. Pre-treatment of acute brain injury induced by i.n dose of PD with TNG orally either 50 or 100 mg/kg for 14 days. (**a**) SOD; (**b**) CAT levels were measured 24h after the last dose of the drug in brain homogenate. Results are expressed as mean ± SEM (n = 8). ^a^Significant difference from normal group *P* < *0.05*. ^b^Significant difference from PD group *P* < *0.05*. ^c^Significant difference from PD + TNG (50 mg/kg) group *P* < *0.05*

PD significantly (*p* < 0.05) increased lipid peroxidation by 68% evidenced by elevated MDA levels in brain homogenates when compared to the control group. However, TNG 50 and 100 mg administration significantly (*p* < 0.05) decreased the elevated MDA levels by 23% and 33%, respectively, as compared to PD treated group. Concerning GSH, SOD, and CAT levels, PD showed a significant (*p* < 0.05) decrease by 55, 46, and 43% when compared to the control group. Nevertheless, TNG 50 and 100 mg administration significantly increased them by 63, 111, 43, 82, and 32% and 74%, respectively, as compared to PD group**.** Moreover, TNG 100 mg/kg returned them to a normal value (Figs. 6 and 7).

### Effect of tangeretin on protein carbonyl levels and DNA fragmentation % in rats received PD induced acute brain injury

**Fig. 8:**
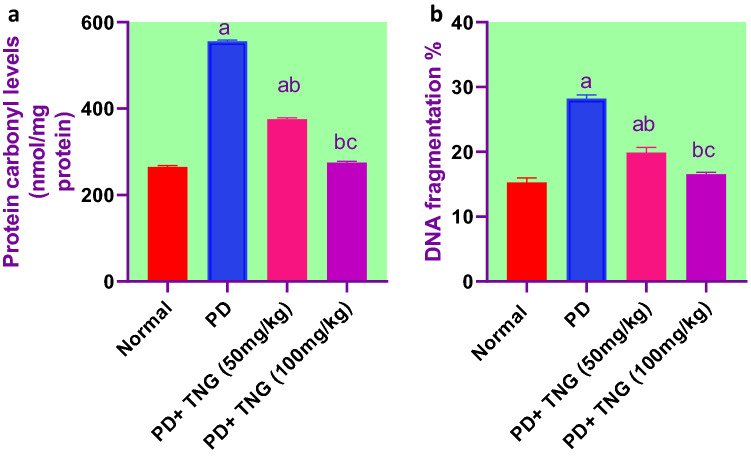
Effect of tangeretin on protein carbonyl levels and DNA fragmentation % in rats received PD induced - acute brain injury. Pre-treatment of acute brain injury induced by i.n dose of PD with TNG orally either 50 or 100 mg/kg for 14 days. (**a**) Protein carbonyl levels; (**b**) DNA fragmentation % were evaluated 24h after the last dose of the drug in brain. Results are expressed as mean ± SEM (n = 8). ^a^Significant difference from normal group *P* < *0.05*. ^b^Significant difference from PD group *P* < *0.05*. ^c^Significant difference from PD + TNG (50 mg/kg) group *P* < *0.05*

Protein carbonyl levels and DNA fragmentation % showed a significant (*p* < 0.05) elevation by 84 and 110%, respectively, in PD group when compared to the control group rats. While, in TNG 50 and 100 mg treatment groups Protein carbonyl levels and DNA fragmentation % significantly (*p* < 0.05) decreased by 32%, 50%, and 30% and 41% respectively, as compared to PD treated group. Moreover, TNG 100 mg/kg returned both to a normal value (Fig. 8).

### Effect of tangeretin on the expression levels of TNF-α and IL-6 in rats received PD induced—acute brain injury

**Fig. 9:**
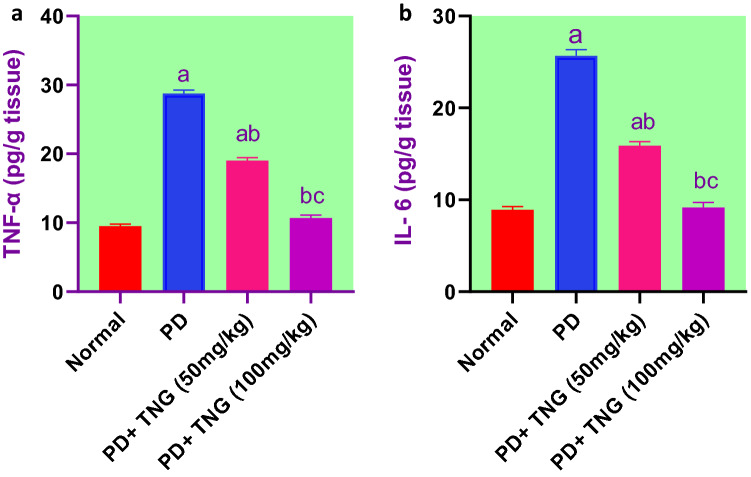
Effect of tangeretin on the expression levels of TNF-α and IL-6 in rats received PD induced - acute brain injury. Pre-treatment of acute brain injury induced by i.n dose of PD with TNG orally either 50 or 100 mg/kg for 14 days. (**a**) TNF-α; (**b**) IL-6 levels were evaluated 24h after the last dose of the drug in brain. Results are expressed as mean ± SEM (n = 8). ^a^Significant difference from normal group *P* < *0.05*. ^b^Significant difference from PD group *P* < *0.05*. ^c^Significant difference from PD + TNG (50 mg/kg) group *P* < *0.05*

PD administration showed a marked (p < 0.05) elevation in TNF-α and IL-6 expression levels by 202 and 188% when compared to the control group rats. On the other hand, rats treated with TNG 50 and 100 mg significantly decreased the expression levels of TNF-α and IL-6 by 34%, 63%, and 38%, 64%, respectively, as compared to PD group. Furthermore, TNG 100 mg/kg returned both to a normal value (Fig. 9).

### Effect of tangeretin on the values of caspase-3 and Nrf2 in the cerebellum of rats received PD induced acute brain injury

Induction of brain injury by PD showed an increment in the value of caspase-3 by twofold with a decrease in Nrf2 value by 91% when compared with control group (*p* < 0.05). Meanwhile, administration of TNG 50 and 100 mg significantly (*p* < 0.05) decreased caspase-3 values by 61, and 75% with an increase in Nrf2 values by one-fold and two-fold respectively, as compared to PD group (table. 2).

**Table 2:** Effect of tangeretin on the values of Caspase-3 and Nrf2 in the cerebellum of rats received PD induced—acute brain injury

Groups	Caspase-3 (% of positive cells/HPF)	Nrf2 (% of positive cells/HPF)
Normal	0.10 ± 0.10	1.10 ± 0.18
PD	2.80^a^ ± 0.13	0.10^a^ ± 0.10
PD + TNG (50 mg/kg)	1.10^ab^ ± 0.27	1.90^ab^ ± 0.18
PD + TNG (100 mg/kg)	0.70^bc^ ± 0.15	2.60^bc^ ± 0.16

Pre-treatment of acute brain injury induced by i.n dose of PD with TNG orally either 50 or 100 mg/kg for 14 days. Levels of caspase-3 and Nrf2 in the cerebellum were measured 24 h after the last dose of the drug. Results are expressed as mean ± SEM (*n* = 8)

^a^Significant difference from normal group *p* < 0.05

^b^Significant difference from PD group *p* < 0.05

^c^Significant difference from PD + TNG (50 mg/kg) group *p* < 0.05

### Effect of tangeretin on the brain histopathological picture in rats received PD induced acute brain injury

**Fig. 10:**
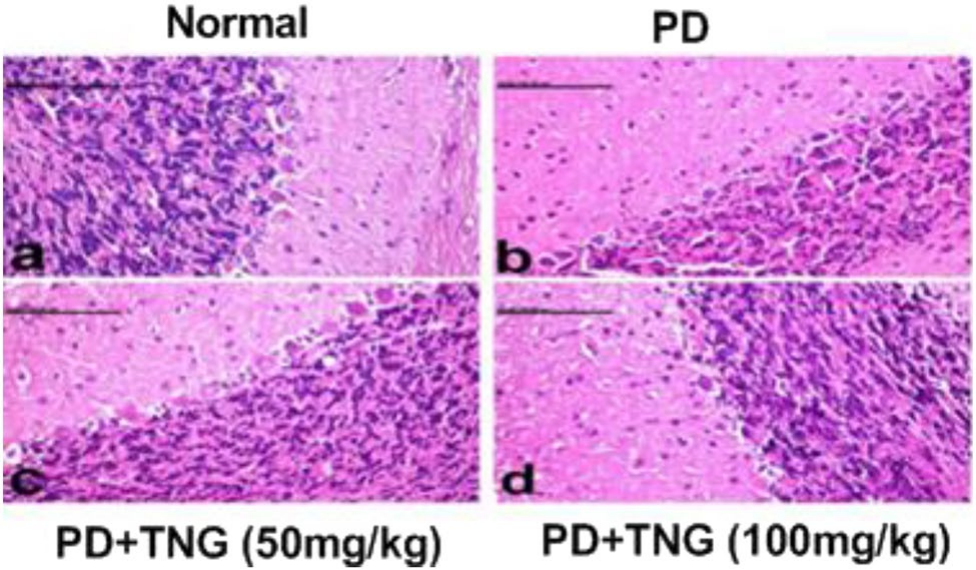
Effect of tangeretin on the histopathological examination of cerebellum of rats received PD induced—acute brain injury. Normal control group showed normal histological structure of the cerebellum with normal Purkinje cells (**a**). PD group revealed shrinkage in the granule layer of cerebellum and necrosis of Purkinje cells with aggregation of microglia around the degenerated neurons (**b**). PD + TNG (50 mg/kg) group showed less scattered degenerated neurons and less shrinkage in granular layer of the cerebellum (**c**). PD + TNG (100 mg/kg) group succeeded to show normal histological structure of the cerebellum (**d**)

Brain sections from the normal control group showed a normal histological picture of the cerebellum with normal Purkinje cells (Fig. 10a). PD-exposed rats showed shrinkage in the granule layer of the cerebellum and necrosis of Purkinje cells with aggregation of microglia around the degenerated neurons (Fig. 10b). Brain sections from PD + TNG (50 mg/kg) group showed less scattered degenerated neurons and less shrinkage in the granular layer of the cerebellum (Fig. 10c). Brain sections from PD + TNG (100 mg/kg) group succeeded to show the normal histological structure of the cerebellum (Fig. 10d).

### Effect of tangeretin on the immunohistochemical values of caspase-3 and NRF-2 in the brain of rats received PD induced acute brain injury

**Fig. 11:**
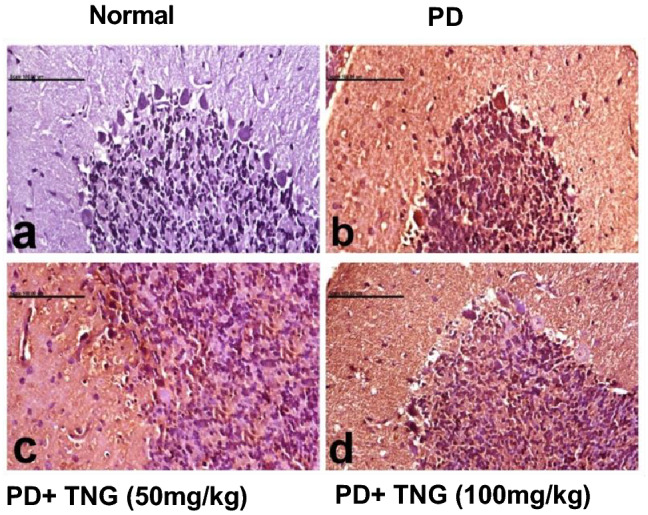
Effect of tangeretin on the expression levels of caspase-3 in the cerebellum of rats received PD induced—acute brain injury. Normal control group revealed no positive stained caspase-3 cells in the cerebellum (**a**), PD group revealed a marked increment in the % of positive stained caspase-3 cells within the granular layer and Purkinje cells of the cerebellum (**b**), PD + TNG (50 mg/kg) group showed a decrement in the % of positive stained caspase-3 cells (**c**), PD + TNG (100 mg/kg) group revealed sparse % in positively stained caspase-3 cells (**d**) (scale bar, 100 µm)

Immunohistochemical staining of the brain tissue with anti-caspase 3 antibodies of the normal control group revealed no positive stained caspase-3 cells in the cerebellum (Fig. 11a). PD exposed group revealed a marked increment in the % of positive stained caspase-3 cells within the granular layer and Purkinje cells of the cerebellum (Fig. 11b). Brain sections of PD + TNG (50 mg/kg) group showed a decrement in the % of positive stained caspase-3 cells (Fig. 11c). Brain sections of PD + TNG (100 mg/kg) group revealed sparse % in positively stained caspase-3 cells (Fig. 11d).

**Fig. 12:**
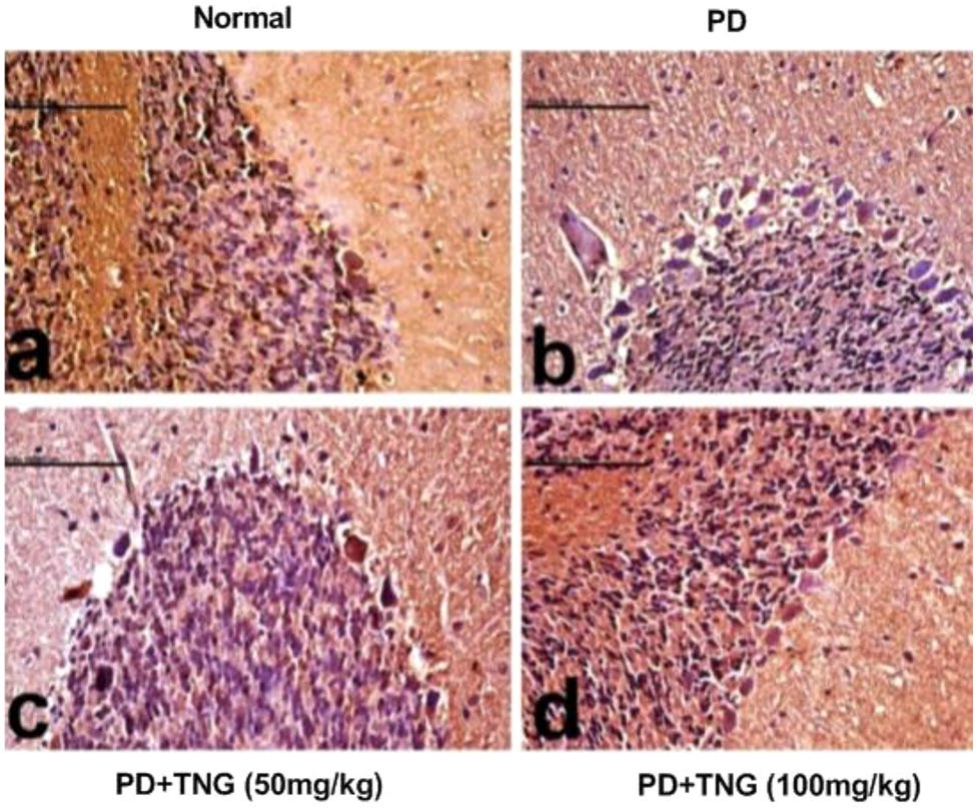
Effect of tangeretin on the expression levels of Nrf-2 in the cerebellum of rats received PD induced—acute brain injury. Normal control group revealed cytoplasmic localization of weakly positive stained Nrf-2 cells in the cerebellum (**a**), PD group revealed a significant decrement in the % of positive stained Nrf-2 cells within the granular layer and Purkinje cells of the cerebellum (**b**), PD + TNG (50 mg/kg) group showed a decrement in the % of positive stained Nrf-2 cells (**c**), PD + TNG (100 mg/kg) group revealed sparse % in positively stained Nrf-2 cells with nuclear staining (**d**) (scale bar, 100 µm)

Immunohistochemical staining of the brain tissue with the anti-Nrf-2 of the normal control group revealed cytoplasmic localization of weakly positive stained Nrf-2 cells in the cerebellum (Fig. 12a). Brain sections of the PD group revealed a significant decrement in the % of positive stained Nrf-2 cells within the granular layer and Purkinje cells of the cerebellum (Fig. 12b). Brain sections of PD + TNG (50 mg/kg) group showed a decrement in the % of positive stained Nrf-2 cells (Fig. 12c). Brain sections of PD + TNG (100 mg/kg) group revealed sparse % in positively stained Nrf-2 cells with nuclear staining (Fig. 12d).

## Discussion

Neuroinflammation, which is a secondary symptom of brain injury, results in neuronal cell death and activation of relocating microglia through the production of chemokines associated with stress and apoptosis. The original injury’s vascular damage permits peripheral immune cells like neutrophils, macrophages, and lymphocytes to enter the body (Alluri et al. 2015). Proinflammatory cytokines secreted by activated microglia, including IL-6, and TNF-α, promote inflammation and apoptotic activity, causing extensive and gradual damage to brain circuitry (Simon et al. 2017). The goal of the current study was to clarify the molecular mechanisms underlying the neuroprotective effects of 0 tangeretin (TNG) on intranasally induced PD-induced brain injury in rats. As a more precise control of the exposure, intranasal instillation of PD solution was used as a route of administration (Horie et al. 2016). Brain injury results in motor coordination and memory impairment in animals as well as in human beings (Walker and Pickett 2007). The current investigation on activity cage and open field tests has shown a significant impairment in motor coordination after PD-induced brain injury. Rat’s motor activity is used to gauge their level of behavioral activity (Tatem et al. 2014). As a result, the decrease in motor activity and coordination of rats demonstrated the extent of neurotoxicity caused by PD. Correspondingly, other studies have reported on the acute neurotoxic effects of PD and how they affect motor control (Salama et al. 2016). The open field results reveal lots of neurobehavioral responses; the frequency of rearing and the number of line crossings are both indicators of activity, exploration, and anxiety.

Anxiety is one of the most prevalent neurobehavioral defects following brain injury. An increased crossing and rearing count in open-field apparatus is believed to be directly correlated with decreased anxiety (Yan et al. 2020). Injured animals showed a high level of anxiety but low explorative and locomotor activity compared to the control animals. Interestingly, it was demonstrated that oral administration of TNG possesses an elevated rate of rearing, motor, and exploratory activities. According to earlier study reports, a notable increase in movement around the field was observed after TNG treatment, suggesting that rats were more motivated and had more exploratory behavior (Yang et al. 2017).

Acetylcholine (ACh) is a crucial neurotransmitter that is involved in learning, memory, and attention. Acutely lowered AChE activity was seen in brain injury patients with chronic cognitive symptoms. Numerous studies have demonstrated a dynamic decline in AChE activity in the brain following brain damage because AChE is an important factor in maintaining AChE levels (Doyle et al. 2016). In the present study, rats exposed to PD showed a decrease in AChE activity but for the first time when TNG was co-administered, this effect was prevented. In line with Moshtaghie et al. 2022 who showed that a single dose of chromium caused a reduction in the levels of acetylcholinesterase activity of various parts of the brain (Moshtaghie et al. 2022). PD causes neurotoxicity by either interfering with AchE synthesis or inhibiting AchE activity by attaching to the anionic active site and restricting the binding of Ach to the enzyme. This causes an excessive buildup of AChE neurotransmitters, which interferes with nerve function and may even be fatal (Kim and Kang 2016).

The probable mechanism by which chromium causes the neurological disease is still up for debate. Previously, it has been reported that transferrin is responsible for the Cr transportation into the circulation and it has a high affinity for Cr and transferrin receptors on the lumen of brain capillaries which mediates the uptake of Cr in the brain (Morris et al. 1987). In the present study, intranasal instillation of PD produced an increase in Cr distribution to the brain. These results indicated that the nasal cavity plays a significant role in the absorption of the instilled dose and direct delivery to the brain and are in line with **(**Mustafa et al. 2016**).** Conversely, TNG was found to change the bio-distribution of Cr and lower its levels in brain tissues.

Decreased ATP content resulted from energy metabolism problems brought on by Cr (VI). Few studies have examined the relationship between reactive oxygen species (ROS) and abnormal mitochondrial metabolism, which may be caused by oxidative stress-mediated mitochondrial apoptosis (Son et al. 2010). A prior study demonstrated that electroplating workers exposed to low levels of occupational Cr (VI) induced- DNA damage (Zhang et al. 2011). The elevation of Cr (VI) inside the cells leads to the production of ROS, which damages the structure and replication of DNA and dysfunction in the transcription genes that control the balance between cell survival and death, implicating them in Cr (VI)-induced apoptosis (Banu et al. 2011). According to results from Abu Zeid et al. 2018, who showed that Cr (VI) increased apoptotic caspase-3, DNA fragmentation, ROS production, and decreased ATP content (Abu Zeid et al. 2018). Concerning activated caspase-3 expression, our present study was linked with an increase in fragmented DNA in the DNA laddering assay. ATP depletion, proteolysis, and oxidative stress usually occur from the huge elevation in excitatory neurotransmitter efflux during brain injury (Biegon 2004). It is well known that a brain injury increases energy requirements because it disturbs ion homeostasis and activates energy-intensive repair processes (Sharma et al. 2009). This is demonstrated by the immediate post-injury increase in glucose utilization seen in cases suffering from brain injury (Marklund et al. 2001). The findings of our study have indicated the beneficial effect of TNG in brain injury by increasing ATP along with decreasing DNA fragmentation in brain tissue and regulation of brain energy. A previous study confirmed our results that citric acid from citrus fruit decreased brain lipid peroxidation, inflammation, and DNA fragmentation (Abdel-SalamOmar et al. 2014).

It has been established that Cr deposition in the mitochondria can potentiate the cascade of lipid peroxidation reactions and cellular damage (Kharel et al. 2016). Prior findings documented that oral exposure to PD can cross freely the blood–brain barrier (BBB), causing dysfunction in the redox homeostasis and triggering the metabolism of several neurodegenetrated proteins (Sun et al. 2015). Antioxidant parameters such as GSH, CAT, and SOD were involved in the endogenous defense against oxidative stress. The cell is protected by these antioxidant enzymes against cell apoptosis induced by oxygen. SOD and CAT catalyze the superoxide anion (O2) transferred to hydrogen peroxide (H2O2) and cause suppression of hydrogen radical generation. Also, GSH can react with ROS regarded as a co-factor with the enzyme GPx to reduce H2O2 and LPO levels. In addition, the MDA level, a marker of lipid peroxidation, can indicate the degree of free radical damage to tissue cells (Grimm et al. 2016). Moreover, an important parameter of oxidative stress is protein carbonylation. Protein carbonylation is a well-documented and quantifiable consequence of oxidative stress in a variety of neuropathologies, including Alzheimer’s and Parkinson’s disease. The role of oxidative stress in the pathology of brain injury has piqued researchers' interest (Ikram et al. 2021). In the experimental study, the administration of TNG could markedly increase the levels of SOD, GSH, and CAT and reduced the MDA and protein carbonyl levels, when compared to the PD group. In agreement with our results, TNG treatment significantly raised the SOD, GSH, and CAT brain contents and reduced the MDA levels (Yang et al. 2020).

Oxidative stress elicits the transcription of antioxidant enzymes by binding the nuclear factor erythroid-2erelated factor-2 (Nrf-2), a chief sensitive antioxidant regulator of redox homeostasis (Yu and Xiao 2021; Yuan et al. 2021). In brain damage, Nrf2 is usually released from the cytoplasm to be translocated into the nucleus to trigger the production of GSH, SOD, and CAT (Lu et al. 2021). Results of the present study showed a significant elevation in the expression of Nrf2 in the cerebellum in TNG treatments, to counteract the brain injury induced by PD intoxication. The current findings supported those of Yan and Huan et al., 2022 who reported that TNG treatment increased the expression of Nrf2 that was reduced in the post-traumatic stress disorder group **(**Yan and Gao 2022**)**.

Activation of astrocytes leads to the production of S100β, a calcium–binding protein that acts on the development and maintenance of the CNS. High levels of S100β could lead to negative effects on the nerve tissue causing apoptosis of glial cells and neurons. The brain injury could lead to the secretion of S100β into the systemic circulation via dysfunction in the BBB **(**Lu et al. 2010**)**. In the current study, S100β protein, a neurotrophic and neuro-biochemical marker useful to detect the noxious effects of PD in the brain and to investigate the role of TNG as a neuro-protective drug. Our findings revealed a significant increment in the values of S100 β in PD-treated rats, as the result of the higher binding capacity of S100β with advanced glycation end products (RAGE) receptor, which enhances the release of cytochrome- c from mitochondria and ROS **(**Niranjan 2014**)**. Interestingly, the present results showed that TNG could ameliorate cellular damage by decreasing S100 β level due to antioxidant activities. Our results are in line with recent findings by Wang et al. 2021 who showed that TNG significantly reduced brain S100β content in rat Alzheimer's model **(**Wang et al. 2021**)**.

Activation of microglial cells within CNS is the first step in inducing neuron inflammation via excessive production of following pro-inflammatory cytokines: IL-6, TNF-α, and adhesion molecules that eventually leads to uncontrolled inflammation in brain injury **(**Block et al. 2007**)**. PD-treated group revealed a dominant increase in IL- 6, and TNF-α, and our data are in the same line with Salama and Elgohary (2021) who reported that i.n. dose of PD creates an inflammatory cascade in Albino Wistar rats **(**Salama and Elgohary 2021**)**. Several agents have been employed for treatment of the brain injury by inhibiting inflammation activity. Pro-inflammatory cytokine expression levels were significantly reduced in the animal groups treated with TNG. Our results markedly decreased the elevated cytokine levels and documented the anti-inflammatory activities of TNG **(**Yang et al. 2020**)**.

Histopathological findings also support the neuro-biochemical indices, where the cerebellum region in the PD group revealed shrinkage in the granule layer of the cerebellum and necrosis of Purkinje cells with aggregation of microglia around the degenerated neurons. In contrast, concurrent treatment of the PD + TNG (50 mg/kg) group showed less scattered degenerated neurons and less shrinkage in granular layer of the cerebellum. Moreover, PD + TNG (100 mg/kg) treated group succeeded to show the normal histological structure of the cerebellum, thus indicating the ameliorating antioxidant, and the anti-inflammatory effect of TNG against PD-induced brain injury.

Our histopathological results clarified that the toxic effects of PD occurred through the generation of ROS as a result of oxidative stress which causes cell and organ damage via mitochondrial dysfunction, decreased oxygen consumption and ATP production, and oxidation of DNA, lipids, and proteins. Interestingly, our immunohistochemical results showed a significant decrement in Nrf2 immunoreactivity in analyzed tissues from PD-treated animals. In contrast, Nrf2 immunoreactivity was increased in the brain tissues of TNG (100 mg/kg) treated rats, whereas limited Nrf2 immunoreactivity was observed in the brain regions of the TNG (50 mg/kg) treated group. Regarding the activated caspase-3 expression, obtained results showed that in PD induced caspase-3 activation when compared to TNG-treated groups. These results are in line with those observed by Wang et al. 2006 who showed Cr(VI) given orally to mice induces hepatocytes apoptosis **(**Wang et al. 2006**)**. These results suggest that in the brain tissue the activation of Nrf2 is an adaptive intracellular response to PD-induced oxidative stress and that Nrf2 is protective against PD-induced apoptosis.

## Conclusion

Our current study demonstrated that tangeretin (TNG) promotes Nrf2-dependent anti oxidative signaling to alleviate potassium dichromate (PD) -induced ROS production. Furthermore, TNG could inhibit PD -triggered inflammation via inhibiting IL- 6, TNF-α, ameliorating BBB dysfunction and apoptosis. Thus, tangeretin might be used as a therapeutic strategy for brain injury and other related diseases.

## Data Availability

Data are available upon request.

## Abbreviations

TNG: Tangeretin
i.n: Intranasal
MDA: Malondialdehyde
GSH: Glutathione
Nrf2: Nuclear factor erythroid 2-related factor 2
TNF-α: Tumor necrosis factor-alpha
IL-6: Interleukin-6
IARC: International agency for research on cancer
K2Cr2O7; PD: Potassium dichromate
ROS: Reactive oxygen species
RNS: Reactive nitrogen species
NRC: National research centre
AChE: Acetylcholinesterase
ACh: Acetylcholine
ATP: Total Adenosine Triphosphate
CAT: Catalase
SOD: Superoxide dismutase
ANOVA: One-way analysis of variance
BBB: Blood–brain barrier
RAGE: Advanced glycation end products
